# Normal-weight visceral obesity promotes a higher 10-year atherosclerotic cardiovascular disease risk in patients with type 2 diabetes mellitus–a multicenter study in China

**DOI:** 10.1186/s12933-023-01876-7

**Published:** 2023-06-12

**Authors:** Jia Zheng, Ye Hu, Hanwen Xu, Yu Lei, Jieji Zhang, Qidong Zheng, Li Li, Weiping Tu, Riqiu Chen, Qiongyao Guo, Xunxiong Zang, Qiaoying You, Zhiyong Xu, Qiang Zhou, Xiaohong Wu

**Affiliations:** 1 Geriatric Medicine Center, Key Laboratory of Endocrine Gland Diseases of Zhejiang Province, Department of Endocrinology, Zhejiang Provincial People’s Hospital (Affiliated People’s Hospital, Hangzhou Medical College), Hangzhou, 310014 Zhejiang People’s Republic of China; 2 Department of Endocrinology, Fenghua District Traditional Chinese Medicine Hospital of Ningbo, Ningbo, 315500 China; 3 Department of Endocrinology, Yuhuan Second People’s Hospital, Taizhou, 317605 China; 4 Department of Endocrinology and Metabolism, Ningbo First Hospital, Ningbo, 315000 China; 5 Department of Endocrinology, Shaoxing Shangyu People’s Hospital, Shaoxing, 312300 China; 6 Department of Endocrinology, Lishui People’s Hospital, Lishui, 323000 China; 7 Department of Endocrinology, The People’s Hospital of Putuo Zhoushan, Zhoushan, 316100 China; 8 Department of Endocrinology, Yueqing People’s Hospital, Wenzhou, 325600 China; 9 Department of Endocrine and Metabolism, Shaoxing People’s Hospital, Shaoxing, 312000 China; 10 Department of Endocrinology, Xianju people’s hospital, Taizhou, 317300 China; 11 Department of Endocrinology, The First Hospital of Jiaxing, Jiaxing, 314000 China

**Keywords:** Normal weight, Visceral obesity, Obesity paradox, Atherosclerotic cardiovascular disease risk, Type 2 diabetes mellitus, Multicentre study

## Abstract

**Background:**

Visceral obesity is associated with high cardiovascular events risk in type 2 diabetes mellitus (T2DM). Whether normal-weight visceral obesity will pose a higher atherosclerotic cardiovascular disease (ASCVD) risk than body mass index (BMI)-defined overweight or obese counterparts with or without visceral obesity remains unclear. We aimed to explore the relationship between general obesity and visceral obesity and 10-year ASCVD risk in patients with T2DM.

**Methods:**

Patients with T2DM (6997) who satisfied the requirements for inclusion were enrolled. Patients were considered to have normal weight when 18.5 kg/m^2^ ≤ BMI < 24 kg/m^2^; overweight when 24 kg/m^2^ ≤ BMI < 28 kg/m^2^; and obesity when BMI ≥ 28 kg/m^2^. Visceral obesity was defined as a visceral fat area (VFA) ≥ 100 cm^2^. Patients were separated into six groups based on BMI and VFA. The odd ratios (OR) for a high 10-year ASCVD risk for different combinations of BMI and VFA were analysed using stepwise logistic regression. Receiver operating characteristic (ROC) curves for diagnosing the high 10-year ASCVD risk were constructed, and areas under the ROC curves were estimated. Potential non-linear relationships between VFA levels and high 10-year ASCVD risk were examined using restricted cubic splines (knot = 4). Multilinear regression was used to identify factors affecting VFA in patients with T2DM.

**Results:**

In patients with T2DM, subjects with normal-weight visceral obesity had the highest 10-year ASCVD risk among the six groups, which had more than a 2-fold or 3-fold higher OR than those who were overweight or obese according to BMI but did not have visceral obesity (all P < 0.05). The VFA threshold for high 10-year ASCVD risk was 90 cm^2^. Multilinear regression showed significant differences in the effect of age, hypertension, drinking, fasting serum insulin, fasting plasma glucose, 2 h postprandial C-peptide, triglyceride, total cholesterol, high-density lipoprotein cholesterol, and low-density lipoprotein cholesterol on VFA in patients with T2DM (all P < 0.05).

**Conclusions:**

T2DM patients with normal-weight visceral obesity had a higher 10-year ASCVD risk than BMI-defined overweight or obese counterparts with or without visceral obesity, which should initiate standardised management for ASCVD primary prevention.

**Supplementary Information:**

The online version contains supplementary material available at 10.1186/s12933-023-01876-7.

## Introduction

Recently, the obesity paradox (OP) has received much attention, especially in cardiovascular disease (CVD), which suggests that patients with different forms of CVD may have an improved outcome if they are overweight or obese (defined by body mass index [BMI]) although they have many more CVD risk factors [1]. This phenomenon was initially described by Gruberg et al. in patients undergoing percutaneous coronary intervention and most likely applied to patients who are overweight and with class I obesity [2]. Subsequent studies have shown a protective role of obesity against overall and cardiovascular mortality in patients with atrial fibrillation, cardiac failure, and coronary artery disease [3]. Possible mechanisms may be related to follow-up, age, BMI, and reverse causation, such as smoking, collider stratification bias, and cardiorespiratory fitness.

Whether OP occurs in patients with type 2 diabetes mellitus (T2DM) remains controversial. Some studies have suggested that individuals who are overweight or obese and have diabetes have lower rates of mortality than those who have normal weight. However, these studies have a number of restrictions owing to their retrospective design and several confounding factors, including smoking habits, antidiabetic treatments, associated pathologies, and lack of data on body fat distribution [4]. Among these, fat distribution, rather than overall adiposity, has attracted much attention. Although BMI is the most commonly used estimator of obesity, it does not capture the distribution of body fat [5]. Coutinho et al. found that visceral obesity was more strongly associated with cardiovascular mortality than BMI [6]. Additionally, compared to peripheral or gluteal-femoral obesity, abdominal fat accumulation is significantly more favourable to coronary disease. Significant CVD risk differences were also detected between visceral and subcutaneous adipose tissue [7, 8]. A prospective study revealed that in Chinese populations with T2DM, all abdominal obesity indexes, including waistline, lipid accumulation product, visceral adiposity index, and Chinese visceral adiposity index, were linked to an elevated risk of CVD events [9].

Normal BMI with increased visceral fat accumulation is relatively common in East Asian populations. Whether normal-weight visceral obesity promotes an increased likelihood of atherosclerotic cardiovascular disease (ASCVD) in patients with T2DM compared to those who have overweight or obesity remains unclear. In this study, we aimed to explore the relationship between general obesity (expressed by BMI) and visceral obesity (expressed by visceral fat area [VFA]) with the 10-year ASCVD risk in T2DM patients without a history of ASCVD, to identify a risk group for early intervention.

## Methods

### Study design and patients

**Fig. 1:**
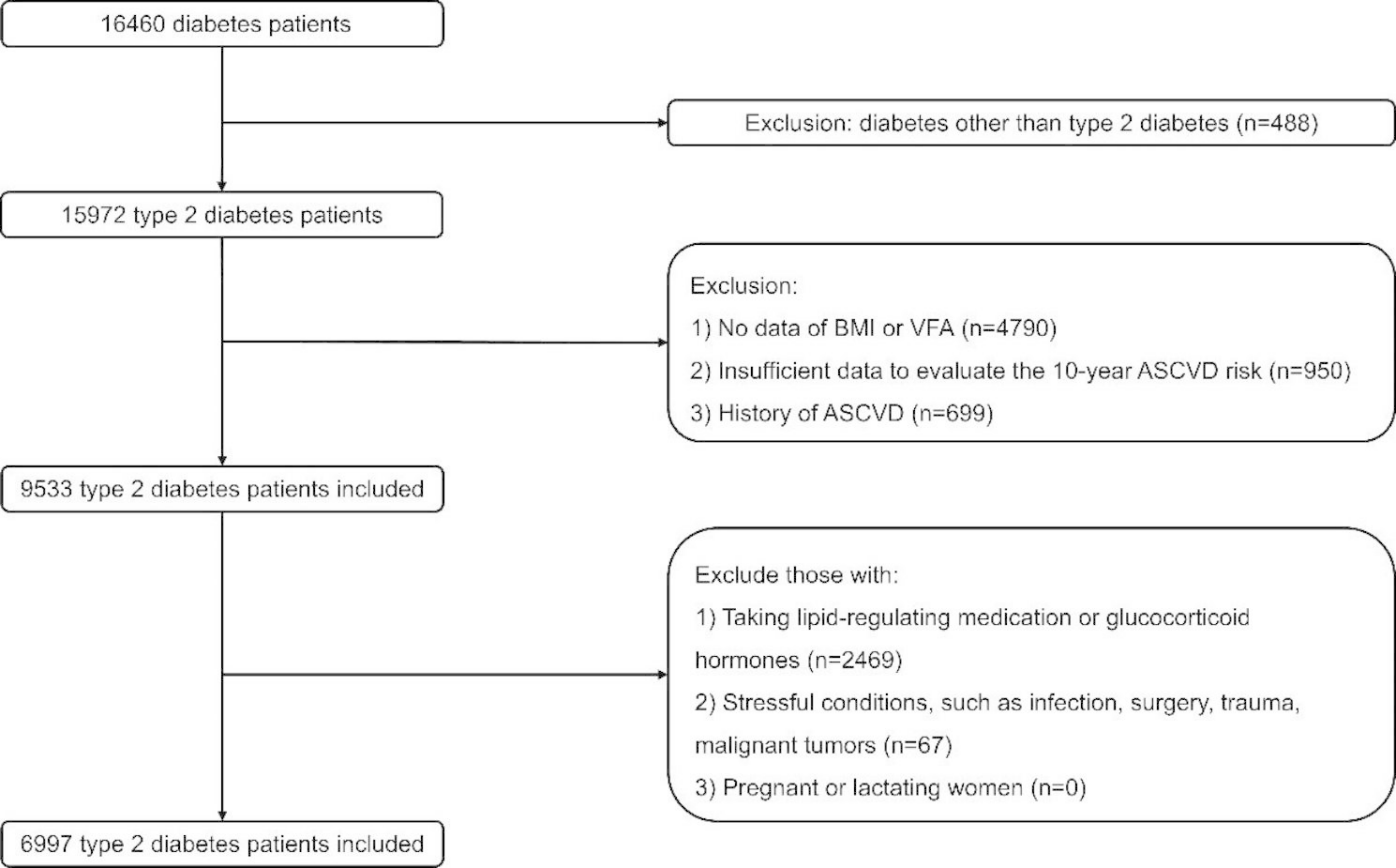
Flow chart of patient recruitment

This was a multicentre retrospective observational study. From April 2020 to April 2022, 16,460 adults from 11 National Metabolic Management Centers (MMC) across East China participated in an observational study of ASCVD risk in T2DM (NCT04866667). All participants underwent the same oral questionnaire interviews, systematic physical examinations, blood sample collection, and abdominal fat area measurements. The exclusion criteria were the following: (1) diabetes other than T2DM (n = 488); (2) missing data of BMI or VFA (n = 4790); (3) missing data required to evaluate the 10-year ASCVD risk (n = 950); (4) history of ASCVD (n = 699); (5) use of lipid-regulating medication or glucocorticoid hormones (n = 2469); and (6) presence of stressful conditions (infection, surgery, trauma, malignant tumours, n = 67). Subsequently, 6997 participants were included for the main analysis (Fig. 1). Participants were considered to have normal weight when 18.5 kg/m^2^ ≤ BMI < 24 kg/m^2^; overweight when 24 kg/m^2^ ≤ BMI < 28 kg/m^2^; and obesity when BMI ≥ 28 kg/m^2^ [10]. Visceral obesity was defined as a VFA ≥ 100 cm^2^ [11]. They were separated into six groups based on different combinations of BMI and VFA.

### Data collection

We collected data on the patients’ general characteristics through a same questionnaire on the day of patient’ visit. To determine the BMI, weight was divided by height squared.

After an 8 h fasting period, blood samples were taken from the patients on the next morning. All laboratory parameters (biochemistry and diabetes-related) in this study were measured with the same method. The homeostasis model assessment of β-cell function (HOMA-β) was calculated as 20 × serum insulin (FINs)/(plasma glucose [FPG] – 3.5), and the homeostasis model assessment of insulin resistance (HOMA-IR) was calculated as FINs × FPG/22.5.

DUALSCAN HDS-2000 was used to perform a bioelectrical impedance study on the VFA and subcutaneous fat area (SFA) [12]. The evaluation procedure was as follows: (1) on the day preceding the check-up, the patients were instructed to start fasting from 20:00 h; (2) the patients were instructed to lie supine with the wrists, ankles, and abdominal skin exposed while the hand and foot electrode clamps and abdominal electrode belt were installed; and (3) the patients were instructed to hold their breath after breathing calmly, and VFA and SFA were measured at that time.

### Definitions

T2DM was diagnosed in accordance with the 2022 American Diabetes Association’s diagnostic criteria of T2DM [13]. Subjects were diagnosed with hypertension if systolic blood pressure (SBP) ≥ 140 mmHg or diastolic blood pressure (DBP) ≥ 90 mmHg or if they were receiving antihypertensive treatments [14].

ASCVD assessment was based on the 2016 Chinese guidelines for the management of dyslipidaemia in adults, in which the 10-year ASCVD risk was evaluated using the 10-year risk assessment model for coronary heart disease and ischaemic CVD in Chinese adults [15, 16]. The 10-year ASCVD risk was stratified into 21 groups according to low-density lipoprotein cholesterol (LDL-C) or total cholesterol (TC) levels, hypertension, and other ASCVD risk factors. The low risk, medium risk, and high risk of ASCVD is separately defined as < 5%, 5–9% and ≥ 10%. Those meeting any of the following criteria were directly classified as high-risk groups: (1) LDL-C ≥ 4.9 mmol/L (190 mg/dl); (2) 1.8 mmol/L (70 mg/dl) ≤ LDL-C < 4.9 mmol/L (190 mg/dl), and patients with diabetes aged 40 years or older (Table 1).

**Table 1:** The assessment process of 10-year ASCVD risk

	Risk factor^*^ (N)	Stratification of TC (mmol/L)		
		3.1 ≤ TC < 4.1	4.1 ≤ TC < 5.2	5.2 ≤ TC < 7.2
		Or 1.8 ≤ LDL-C < 2.6	Or 2.6 ≤ LDL-C < 3.4	Or 3.4 ≤ LDL-C < 4.9
**No hypertension**	**0–1**	Low risk (< 5%)	Low risk (< 5%)	Low risk (< 5%)
	**2**	Low risk (< 5%)	Low risk (< 5%)	Medium risk (5-9%)
	**3**	Low risk (< 5%)	Medium risk (5-9%)	Medium risk (5-9%)
**Hypertension**	**0**	Low risk (< 5%)	Low risk (< 5%)	Low risk (< 5%)
	**1**	Low risk (< 5%)	Medium risk (5-9%)	Medium risk (5-9%)
	**2**	Medium risk (5-9%)	High risk (≥ 10%)	High risk (≥ 10%)
	**3**	High risk (≥ 10%)	High risk (≥ 10%)	High risk (≥ 10%)

*Risk factors: smoking, low HDL-C and male over 45 years old (female over 55 years old)

### Statistical analysis

Data not normally distributed was summarised utilising medians and quartiles, while categorical variables were described as proportions. To identify any distinctions between the six groups, the Kruskal–Wallis rank-sum test was used for multiple samples. The chi-squared test was used to examine discrepancies in categorical variables. The odds ratios (OR) for a high 10-year ASCVD risk for different combinations of BMI and VFA was analysed using stepwise logistic regression. Receiver operating characteristic (ROC) curves for diagnosing the high 10-year ASCVD risk were constructed, and areas under the ROC curves (AUC) were estimated. Potential non-linear relationships between VFA levels and high 10-year ASCVD risk were examined using restricted cubic splines (knot = 4). A heatmap was made to depict the correlation between other variables and VFA in all patients with T2DM. Multilinear regression was used to identify factors affecting VFA in patients with T2DM. SPSS (IBM, version 25.0), MedCalc (version 20.0.4), R (version 3.6), and Prism (GraphPad, version 9.0) were employed for all the analyses. P < 0.05 was deemed statistically significant.

## Results

### Baseline characteristics

The baseline characteristics of patients with T2DM based on different combinations of BMI and VFA are shown in Table 2. According to the BMI of 6997 patients with T2DM, 2860 (40.9%) had normal weight, 2980 (42.6%) were overweight, and 1157 (16.5%) were obese. Simultaneously, 2777 (39.7%) patients with T2DM were found to have visceral obesity based on the VFA. The results of statistical analysis showed that sex, age, BMI, VFA, SFA, SBP, DBP, hypertension, smoking, drinking, glycosylated haemoglobin A_1c_ (HbA_1c_), FPG, 2 h postprandial plasma glucose (2 h-PPG), FINs, 2 h postprandial serum insulin (2 h-PINS), fasting C-peptide (FCP), 2 h postprandial C-peptide (2 h-PCP), HOMA-IR, HOMA-β, triglyceride (TG), TC, high-density lipoprotein cholesterol (HDL-C), LDL-C, creatinine (Cr), alanine aminotransferase (ALT), aspartate aminotransferase (AST), and gamma glutamyl-transpeptidase (γ-GT) levels were significantly different among the six groups (all P < 0.05).

**Table 2:** Baseline characteristics of the study populations (N = 6997) according to different combinations of BMI and VFA

					Normal Weight		Overweight		Obesity		*P* value
Demographic variables					Normal	Visceral obesity	Normal	Visceral obesity	Normal	Visceral obesity	
		Participants			2531(36.2%)	329(4.7%)	1495(21.4%)	1485(21.2%)	194(2.8%)	963(13.8%)	NA
		Males			1456(57.5%)	244(74.2%)	955(63.9%)	1041(70.1%)	111(57.2%)	621(64.5%)	< 0.001
		Age, yr			55.00(47.00, 62.00)	57.00(49.00, 62.00)	55.00(48.00, 61.00)	55.00(48.00, 63.00)	54.00(40.00, 62.00)	55.00(48.00, 62.00)	< 0.001
		Body mass index, kg/m2			22.13(20.89, 23.13)	23.09(22.37, 23.55)	25.39(24.65, 26.23)	26.12(25.15, 26.98)	29.08(28.54, 30.74)	30.13(28.89, 32.52)	< 0.001
		Visceral fat area, cm2			61.00(44.00, 77.00)	111.00(104.00, 121.85)	81.40(69.00, 91.00)	119.25(108.20, 135.03)	86.80(78.00, 95.00)	144.70(122.00, 171.70)	< 0.001
		Subcutaneous fat area, cm2			122.00(100.13, 145.00)	151.10(132.00, 171.45)	168.00(147.00, 193.70)	185.55(164.00, 210.00)	229.40(203.63, 278.18)	252.00(217.00, 301.90)	< 0.001
		Systolic blood pressure, mmHg			123.00(113.00, 136.00)	129.00(118.50, 144.00)	129.00(119.00, 141.00)	130.00(120.00, 141.00)	133.00(123.00, 141.00)	135.00(125.00, 147.00)	< 0.001
		Diastolic blood pressure, mmHg			73.00(66.00, 80.00)	76.00(68.00, 83.00)	75.00(68.00, 82.00)	77.00(70.00, 85.00)	78.00(70.00, 86.00)	80.00(73.00, 88.00)	< 0.001
		Hypertension			839(33.1%)	160(48.6%)	651(43.5%)	770(51.9%)	89(45.9%)	616(64.0%)	< 0.001
		Smoking			727(28.7%)	133(40.4%)	473(31.6%)	581(39.1%)	34(17.5%)	312(32.4%)	< 0.001
		Drinking			672(30.9%)	127(50.4%)	439(33.3%)	556(44.5%)	27(15.6%)	327(38.9%)	< 0.001
		Family history of diabetes			1086(42.9%)	142(43.2%)	625(41.8%)	626(42.2%)	82(42.3%)	384(39.9%)	0.724
**Laboratory variables**											
	Glycosylated haemoglobin A_1c_, mmol/mol				63.93(47.54, 87.07)	68.31(51.91, 87.98)	62.84(48.63, 85.25)	65.03(51.91, 84.70)	59.02(45.68, 85.25)	7.80(61.75, 80.33)	0.025
	Glycosylated haemoglobin A_1c_, %				8.00(6.50, 10.30)	8.40(6.90, 10.20)	7.90(6.60, 9.95)	8.10(6.90, 9.90)	7.55(6.33, 9.95)	7.80(6.60, 9.50)	0.025
	Fasting blood glucose, mmol/L				7.71(6.12, 10.31)	7.95(6.32, 10.28)	8.00(6.51, 10.65)	8.16(6.69, 10.52)	7.47(6.02, 10.14)	7.85(6.49, 9.84)	< 0.001
	2 h postprandial blood glucose, mmol/L				15.14(10.94, 19.32)	15.27(11.57, 19.51)	15.10(11.21, 18.90)	14.71(11.32, 18.00)	13.25(8.88, 17.66)	14.09(10.81, 17.85)	< 0.001
	Fasting insulin, µIU/mL				5.80(3.10, 10.30)	6.50(3.70, 10.92)	8.40(5.10, 14.14)	8.70(5.76, 13.70)	13.89(7.47, 29.83)	11.73(7.07, 19.54)	< 0.001
	2 h postprandial insulin, µIU/mL				21.97(11.70, 39.78)	24.44(14.62, 45.13)	31.24(16.60, 54.94)	30.61(18.26, 52.88)	40.10(23.24, 117.99)	38.70(21.80, 73.80)	< 0.001
	Fasting C-peptide, ng/mL				0.44(0.16, 1.46)	1.30(0.32, 2.10)	0.37(0.19, 1.85)	1.70 (0.29, 2.59)	0.33(0.22, 1.76)	1.10(0.27, 2.75)	< 0.001
	2 h postprandial C-peptide, ng/mL				1.40(0.46, 3.90)	3.86(0.88, 5.60)	1.13(0.48, 4.82)	4.03(0.76, 6.56)	1.02(0.50, 3.98)	2.60(0.68, 6.61)	< 0.001
	Homeostatic model assessment of insulin resistance				2.13(1.09, 4.01)	2.45(1.25, 4.14)	3.19(1.74, 5.60)	3.30(2.08, 5.50)	4.67(2.46, 13.31)	4.36(2.37, 7.45)	< 0.001
	Homeostasis model assessment of β-cell function				26.07(11.92, 56.39)	25.42(13.51, 50.69)	37.07(19.97, 72.95)	37.15(20.67, 66.18)	77.47(29.46, 201.27)	50.82(29.02, 99.94)	< 0.001
	Triglyceride, mg/dL				1.21(0.85, 1.82)	1.68(1.16, 2.47)	1.45(1.03, 2.13)	1.75(1.23, 2.57)	1.52(1.12, 2.30)	1.73(1.24, 2.54)	< 0.001
	Total cholesterol, mg/dL				4.72(4.08, 5.42)	5.06(4.28, 5.70)	4.79(4.17, 5.50)	4.89(4.27, 5.72)	4.89(4.14, 5.61)	4.91(4.22, 5.64)	< 0.001
	High density lipoprotein cholesterol, mg/dL				1.23(1.03, 1.47)	1.13(0.97, 1.30)	1.14(0.97, 1.38)	1.08(0.92, 1.28)	1.10(0.95, 1.27)	1.11(0.95, 1.28)	< 0.001
	Low density lipoprotein cholesterol, mg/dL				2.68(2.16, 3.29)	2.77(2.28, 3.33)	2.75(2.22, 3.34)	2.79(2.26, 3.36)	3.07(2.44, 3.51)	2.86(2.22, 3.50)	< 0.001
	Serum creatinine, mg/dL				60.50(51.00, 72.10)	66.00(56.00, 80.60)	61.40(51.95, 73.55)	67.00(56.00, 79.20)	64.10(50.00, 76.00)	66.00(54.00, 78.00)	< 0.001
	Alanine aminotransferase, IU/L				19.00(14.00, 29.00)	25.00(16.00, 41.50)	23.00(17.00, 35.00)	27.00(18.00, 41.00)	30.50(21.00, 50.88)	31.00(20.00, 50.00)	< 0.001
	Aspartate aminotransferase, IU/L				19.00(15.13, 25.00)	21.00(17.00, 30.00)	20.00(16.75, 26.00)	21.00(16.98, 28.00)	23.20(19.00, 35.83)	24.00(18.00, 35.00)	< 0.001
	Gamma glutamyl-transpeptidase, IU/L				22.00(15.00, 36.00)	33.00(20.00, 60.50)	26.00(18.00, 43.00)	34.00(23.00, 55.00)	34.00(20.02, 56.75)	37.00(24.00, 61.00)	< 0.001

Data are presented as median (IQR) or n (%)

Patients were considered to have normal weight when 18.5 kg/m^2^ ≤ BMI < 24 kg/m^2^; overweight when 24 kg/m^2^ ≤ BMI < 28 kg/m^2^; and obesity when BMI ≥ 28 kg/m^2^. Visceral obesity was defined as VFA ≥ 100 cm^2^

### Association between the high 10-year ASCVD risk and BMI/VFA status in the background of T2DM

**Fig. 2:**
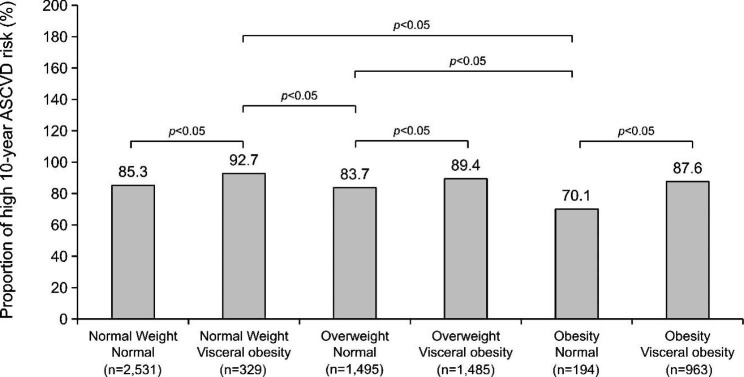
The proportion of high 10-year ASCVD risk according to BMI/VFA status in patients with T2DM. Patients were considered to have normal weight when 18.5 kg/m^2^ ≤ BMI < 24 kg/m^2^; overweight when 24 kg/m^2^ ≤ BMI < 28 kg/m^2^; and obesity when BMI ≥ 28 kg/m^2^. Visceral obesity was defined as VFA ≥ 100 cm^2^

**Fig. 3:**
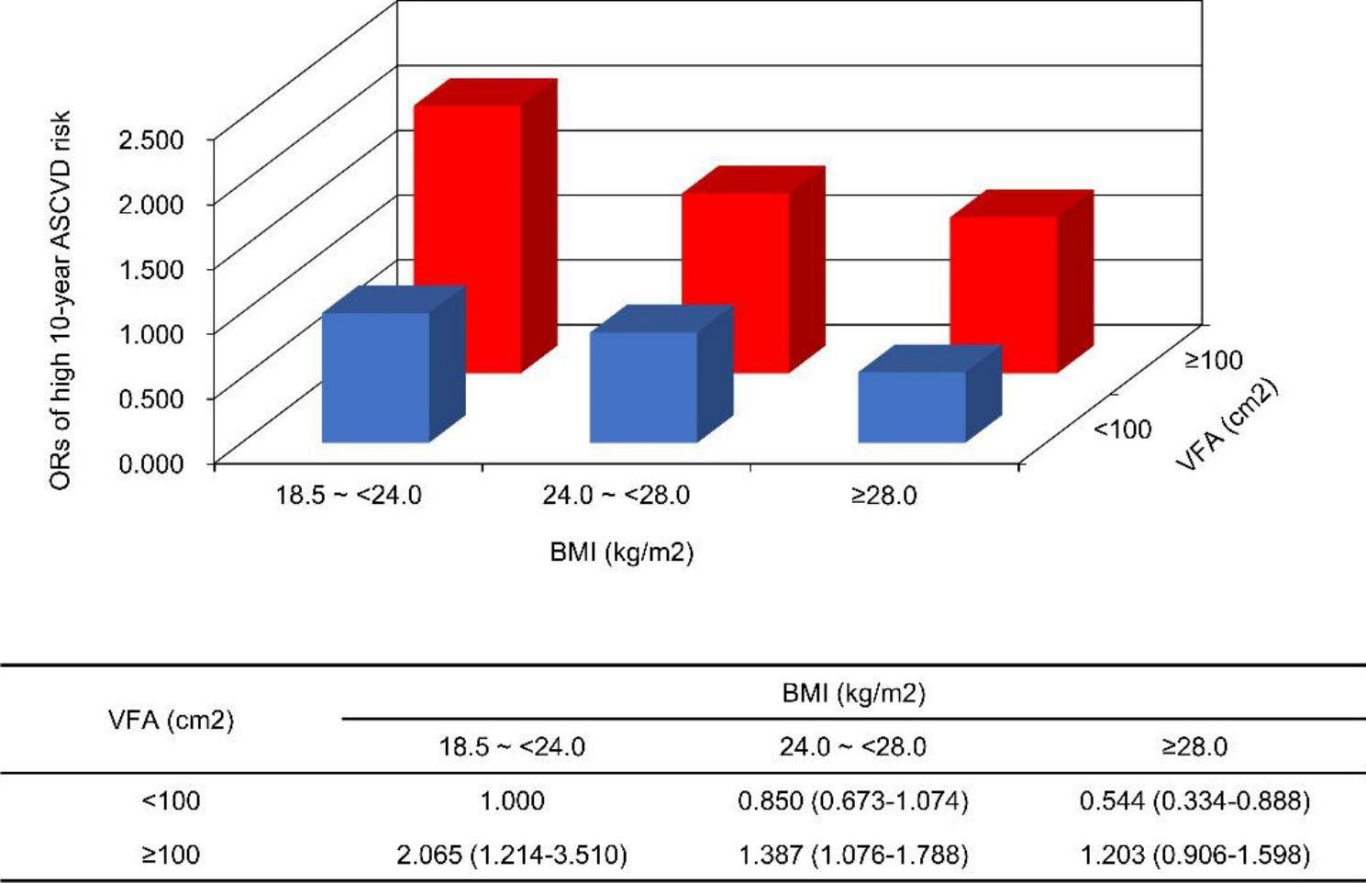
ORs and 95% CIs for high 10-year ASCVD risk according to BMI/VFA status in T2DM patients. The results were adjusted by age and sex

**Fig. 4:**
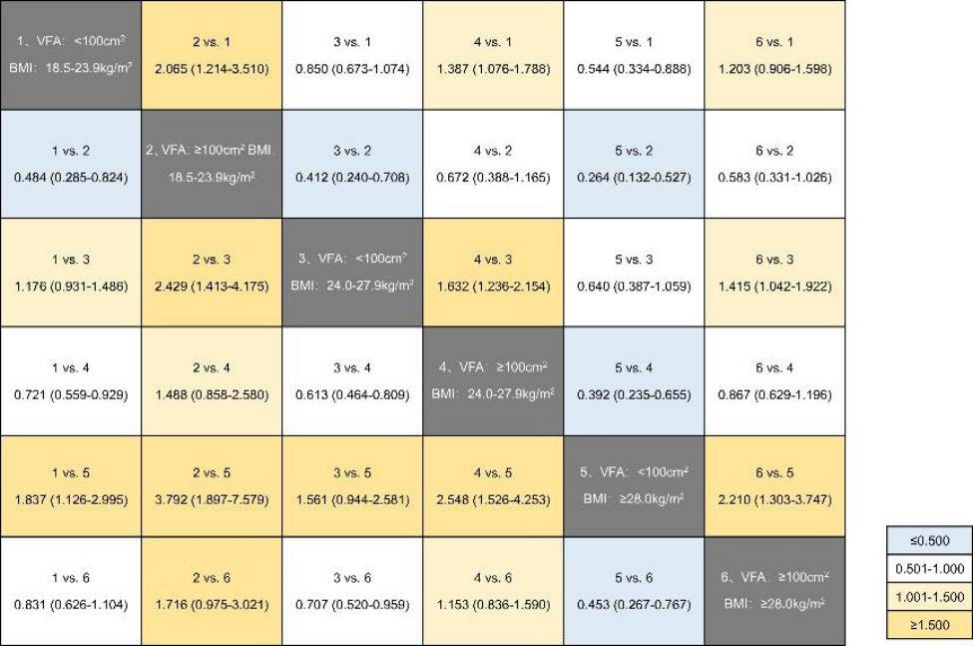
ORs and 95% CIs for high 10-year ASCVD risk according to BMI/VFA status in T2DM patients. The results were adjusted by age and sex

The association between the high 10-year ASCVD risk and BMI/VFA status is shown in Figs. 2 and 3, and 4. In patients with T2DM, subjects with visceral obesity had a higher 10-year ASCVD risk than those with normal visceral fat area, regardless of the BMI category (all P < 0.05). Of note, patients with normal-weight visceral obesity had the highest 10-year ASCVD risk among the six groups, which had more than a 2-fold or 3-fold higher OR than those who were overweight or obese according to BMI but did not have visceral obesity (OR = 2.429, 95% confidence interval [CI]: 1.413–4.175, P = 0.001; OR = 3.792, 95% CI: 1.897–7.579, P < 0.001).

### Comparison of BMI, VFA, and BMI + VFA for diagnosing the high 10-year ASCVD risk in patients with T2DM

**Fig. 5:**
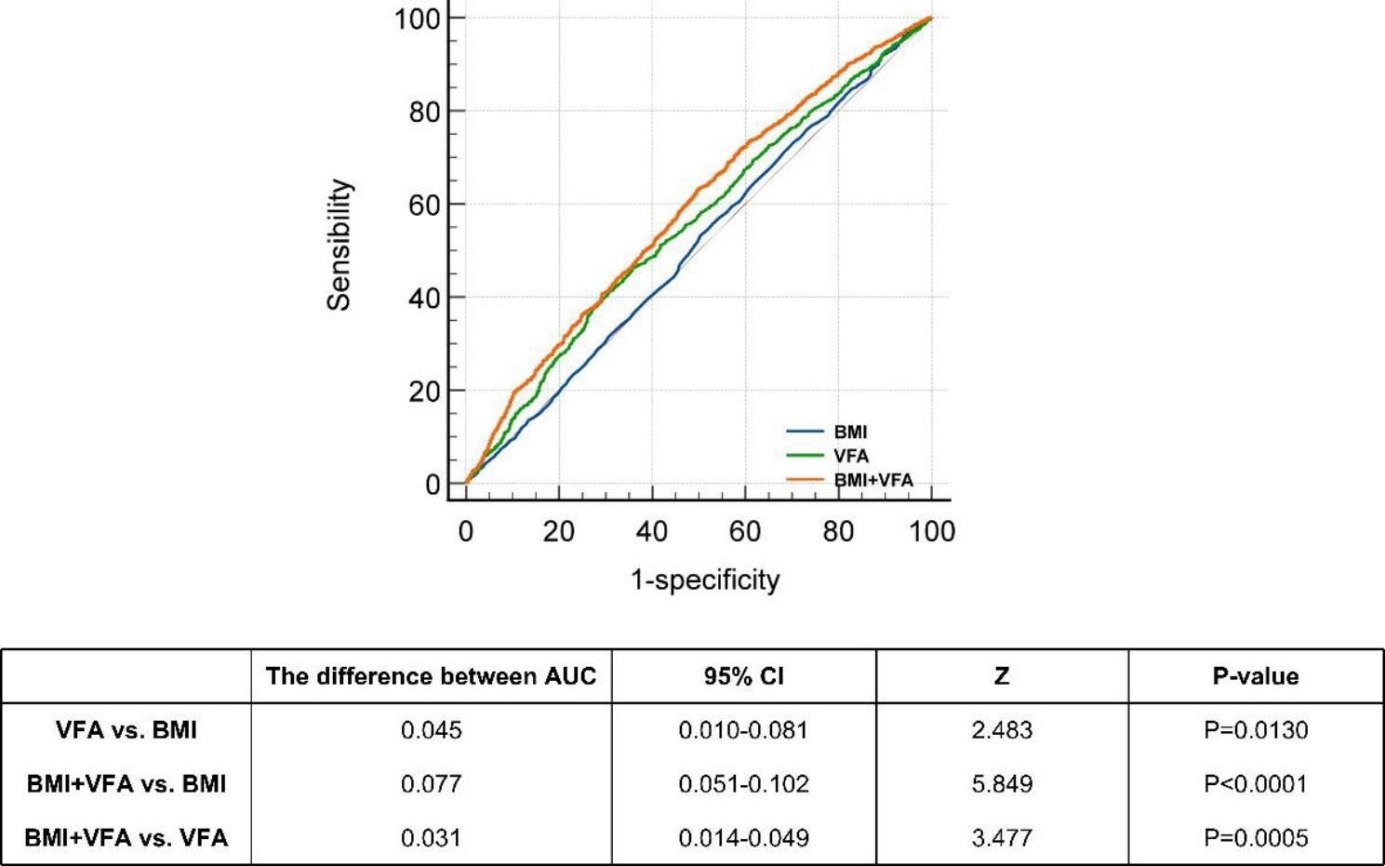
The calculation and comparison of AUCs for diagnosing high 10-year ASCVD risk in T2DM patients. The AUC of BMI, VFA, and BMI + VFA is respectively 0.511 (95% CI: 0.499–0.523), 0.556 (95% CI: 0.554–0.568) and 0.588 (95% CI: 0.576–0.599)

**Fig. 6:**
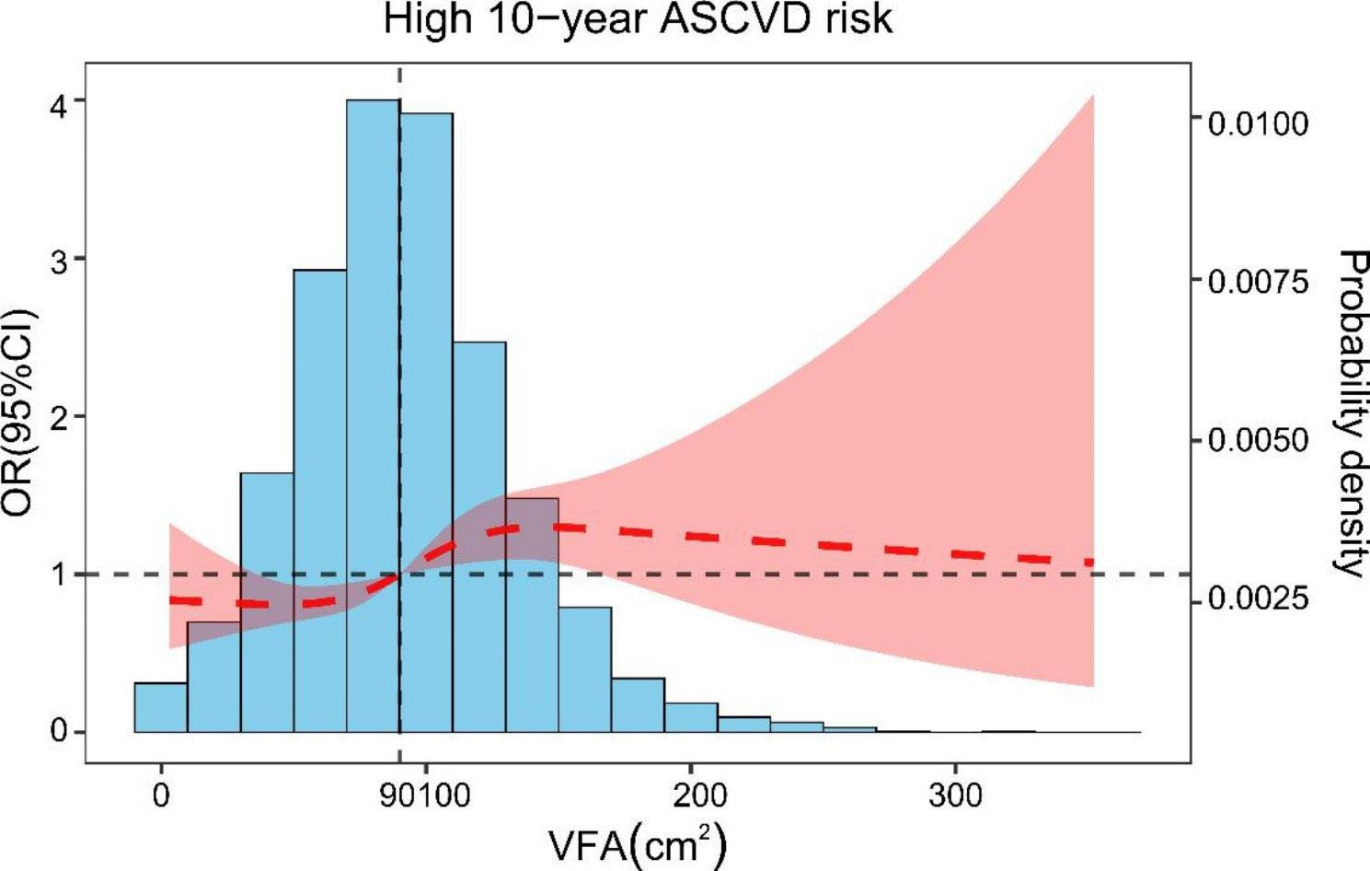
The association between VFA and a high 10-year ASCVD risk in T2DM patients. The result was adjusted by age and sex

There were 6021 patients with T2DM with (86.1%) high 10-year ASCVD risk and 976 with (13.9%) non-high-risk. The ROC curves and AUCs for BMI, VFA, and BMI + VFA are shown in Fig. 5; Table 3. The AUC of BMI, VFA, and BMI + VFA was respectively 0.511 (95% CI: 0.499–0.523), 0.556 (95% CI: 0.554–0.568), and 0.588 (95% CI: 0.576–0.599). In addition, the difference in AUC between VFA and BMI was 0.045 (Z = 2.483, P = 0.0130), between BMI + VFA and BMI was 0.077 (Z = 5.849, P < 0.0001), and between BMI + VFA and VFA was 0.031 (Z = 3.477, P = 0.0005). The association between VFA and a high 10-year ASCVD risk fitted a non-linear spline model (Fig. 6). When VFA was ≥ 90 cm^2^, the ORs for high 10-year ASCVD risk was ≥ 1. When VFA was ≥ 100 cm^2^, the high 10-year ASCVD risk first increased and then decreased.

**Table 3:** The AUCs of BMI, VFA, and BMI + VFA for diagnosing the high 10-year ASCVD risk in T2DM patients

	AUC	95% CI	Youden Index	Sensibility	Specificity
**BMI**	0.511	0.499–0.523	0.029	73.180	29.710
**VFA**	0.556	0.544–0.568	0.104	41.270	69.160
**BMI + VFA**	0.588	0.576–0.599	0.133	62.780	50.510

### Association between the other variables and VFA in patients with T2DM

**Fig. 7:**
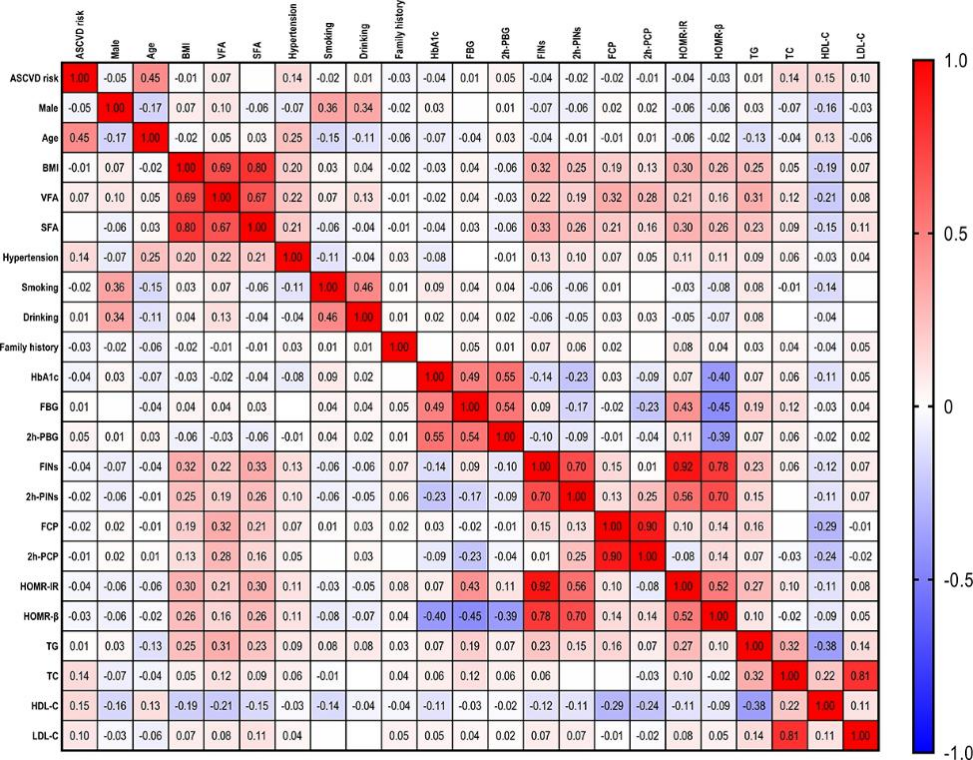
The heatmap depicts the correlational relationships between the other variables and VFA in T2DM patients

The correlation analysis of all patients with T2DM revealed that VFA was positively correlated with sex, age, hypertension, smoking, drinking, FINs, 2 h-PINs, FCP, 2 h-PCP, TG, TC and LDL-C and negatively correlated with HDL-C in Spearman’s analyses (all, P < 0.05; Fig. 7). Multilinear regression analysis showed significant differences in the effect of age (B = 0.202, t = 3.837, P < 0.001), hypertension (B = 17.070, t = 14.918, P < 0.001), drinking (B = 9.812, t = 7.491, P < 0.001), FINs (B = 0.057, t = 2.456, P = 0.014), FCP (B = 5.838, t = 14.278, P < 0.001), 2 h-PCP (B = 0.284, t = 2.424, P = 0.015), TG (B = 3.716, t = 9.144, P < 0.001), TC (B = 1.531, t = 2.147, P = 0.032), HDL-C (B = -9.469, t = -6.141, P < 0.001), and LDL-C (B = 2.513, t = 2.955, P = 0.003) on VFA in patients with T2DM (Table 4).

**Table 4:** A multilinear regression model of VFA in T2DM patients

Multilinear Regression	R	0.446	R^2^	0.199	Adjusted R^2^	0.197	Tolerance	VIF
	Variables	B	SE	β	t	Sig.		
	**Constant**	49.009	4.220		11.614	< 0.001		
	**Male**	1.345	1.227	0.015	1.096	0.273	0.857	1.167
	**Age**	0.202	0.053	0.052	3.837	< 0.001	0.916	1.091
	**Hypertension**	17.070	1.144	0.200	14.918	< 0.001	0.936	1.069
	**Smoking**	1.467	1.359	0.016	1.080	0.280	0.771	1.298
	**Drinking**	9.812	1.310	0.110	7.491	< 0.001	0.776	1.289
	**Fasting insulin**	0.057	0.023	0.038	2.456	0.014	0.707	1.414
	**2 h postprandial insulin**	0.009	0.007	0.021	1.323	0.186	0.697	1.434
	**Fasting C-peptide**	5.838	0.409	0.218	14.278	< 0.001	0.722	1.385
	**2 h postprandial C-peptide**	0.284	0.117	0.036	2.424	0.015	0.748	1.337
	**Triglyceride**	3.716	0.406	0.144	9.144	< 0.001	0.685	1.460
	**Total cholesterol**	1.531	0.713	0.044	2.147	0.032	0.396	2.527
	**High density lipoprotein cholesterol**	-9.469	1.542	-0.090	-6.141	< 0.001	0.789	1.267
	**Low density lipoprotein cholesterol**	2.513	0.850	0.056	2.955	0.003	0.463	2.160

Abbreviations: SE, stand error; Sig, significance. VIF, variance inflation factor: an indicator for diagnosis of collinearity

## Discussion

Our analyses of data from 11 MMCs across East China showed that patients with T2DM with normal-weight visceral obesity had a higher 10-year ASCVD risk than those with BMI-defined overweight or obesity, with or without visceral obesity. In addition, VFA was more suggestive of ASCVD risk than BMI, and the threshold for a high 10-year ASCVD risk was 90 cm^2^. Moreover, we discovered that age, hypertension, drinking, FINs, FCP, 2 h-PCP, TG, TC, HDL-C, and LDL-C levels influenced VFA. Our findings suggest that T2DM patients with normal-weight visceral obesity should initiate standardised management for the primary prevention of ASCVD.

To our knowledge, this is the first study to show that normal-weight visceral obesity, as measured by a combination of BMI and VFA, is associated with a higher 10-year ASCVD risk in patients with T2DM. This warns us that when assessing the ASCVD risk in patients with T2DM, we should pay special attention to those with normal-weight visceral obesity. In addition, it was suggested that conventional and single screening by BMI was no longer adequate for the accurate assessment of obesity, whereas VFA was more suggestive. Furthermore, the threshold for VFA suggestive of a high 10-year ASCVD risk was 90 cm^2^. Previous studies have linked elevated VFA levels to an elevated risk of CVD in individuals with T2DM [17, 18]. Summarising the results of several cardiometabolic imaging studies, Piche et al. [19] found that some normal weight or overweight people with excessive visceral adipose tissue were at high risk, which is usually accompanied by fat build-up in normal lean tissue. Excess ectopic fat and visceral adipose tissue significantly determine the risk of CVD [20, 21], while subcutaneous adipose tissue is linked to retained insulin sensitivity and reduces the risk of metabolic diseases [22–24]. Thus, BMI and VFA should be coupled for the assessment of obesity.

We attempted to explore the factors associated to VFA. After correcting for sex and age, we found that hypertension; the degree of alcohol consumption; and the levels of FINs, FCP, 2 h-PCP, TG, TC, and LDL-C were positively correlated to the levels of VFA. The lower the HDL-C levels, the higher the VFA levels. Age is not considered as an independent criterion in current adult obesity assessment guidelines [25]. However, age-related body composition changes include visceral fat, reduced bone mineral density, and sarcopenia [26]. Results from previous research regarding the connection between alcohol consumption and overweight/obesity are inconsistent. A recent study that analysed 127 large cohorts found that when comparing between light alcohol drinkers or non-alcohol drinkers, heavy alcohol drinkers had a higher risk of developing abdominal obesity [27], which is consistent with our findings. Several epidemiological investigations have indicated that accumulation of VFA is linked with the risk of hypertension, insulin resistance, and dyslipidaemia [28–30]. Our research found that insulin resistance was related to visceral obesity. Kolb et al. [31, 32] have provided support for the obesity-promoting role of insulin. Dyslipidaemia, which is characterised by increased LDL-C and TG levels and reduced HDL-C levels, has been established as a characteristic of cardiovascular and obesity diseases [33, 34]. Zhu et al. [35] found that participants with central obesity residing in Shanghai Suburban had higher levels of TG, TC, and LDL-C and lower levels of HDL-C, which is consistent with our results. Therefore, controlling blood pressure, reducing alcohol intake, and treating insulin resistance and dyslipidaemia early in these patients can reduce obesity and CVD risk.

Visceral obesity can be assessed using various methods including computed tomography (CT), magnetic resonance imaging (MRI), bioelectrical impedance analysis, and anthropometric indicators. CT is the gold standard for measuring visceral adipose tissue, which can be done quickly and analysed using a commercial software. However, it involves exposure to radiation; therefore, it is not suitable for continuous assessment over time or for the assessment of changes after an intervention. MRI does not involve radiation and can be used for continuous assessment over time; however, is time-consuming and expensive [36, 37]. Anthropometric indicators are easy to measure but do not correlate well with direct imaging-based assessments of visceral obesity. CT and MRI provide greater sensitivity and specificity for measuring VFA [37]. In our study we employed, bioelectrical impedance analysis, which is a non-invasive, convenient, and accurate method. Omura-Ohata et al. [38] revealed that bioelectrical impedance analysis could be an alternative to CT as a non-intrusive and inexpensive method for assessing VFA in patients with diabetes. Moreover, Park et al. [39] found that dual abdominal bioelectrical impedance analysis performed on a DUALSCAN HDS-2000 machine was more precise in determining abdominal VFA than whole-body bioelectrical impedance analysis referenced to CT.

Over the last few decades, several widely recognised algorithms and models for assessing ASCVD risk have been created and revised in the USA and Europe; one of the most widely used is the Pooled Cohort Equations (PCEs) of the 2013 American College of Cardiology/American Heart Association assessment of cardiovascular risk guidelines [40]. However, this does not perform well in East Asian populations [41, 42]. Recent research has shown that the ischaemic CVD risk indicated by the Chinese model is lower than the 10-year ASCVD risk predicted by PCEs [43, 44]. The 10-year risk assessment model for coronary heart disease and ischaemic CVD in Chinese adults, combined with the spectrum of diseases and prevalence of cardiovascular risk factors in China, has been widely used in a large number of studies [45, 46]. Therefore, we chose the Chinese model to evaluate the 10-year ASCVD risk in the patients in our study [16].

This study had several limitations. First, owing to the study’s cross-sectional design, we were unable to investigate the long-term dynamic connection between obesity status and ASCVD risk. Second, we could not assess the impact of therapeutic interventions, such as lifestyle changes and medications, on the risk of ASCVD in individuals with normal-weight visceral obesity. However, the long-term follow-up of the patients included in our study is ongoing.

## Conclusion

Our study found that T2DM patients with normal-weight visceral obesity had a higher 10-year ASCVD risk than individuals with T2DM and BMI-defined overweight or obesity, with or without visceral obesity, which should initiate standardised management for ASCVD primary prevention. This may have significant clinical implications because most people do not consider patients with T2DM with normal BMI and visceral obesity as a priority population for ASCVD primary prevention. Future research should focus on identifying factors associated with the development of normal-weight visceral obesity and enhancing our understanding in the impact of normal-weight visceral obesity on health outcomes. In this regard, the use of a combined BMI and VFA measure may provide a better stratification of obesity-related risk factors in clinical practice than relying solely on either method alone.

## Electronic supplementary material

Below is the link to the electronic supplementary material.

**Additional file 1: Figure S1** The proportion of high 10-year ASCVD risk according to BMI/VFA status in male patients with T2DM. **Figure S2.** The proportion of high 10-year ASCVD risk according to BMI/VFA status in female patients with T2DM. **Table S1**. Association between the other variables and VFA in T2DM patients. **Table S2.** Linear regression analysis of VFA in T2DM patients

## Data Availability

The data from this study are available from the corresponding author upon reasonable request. The authors declare that they have no conflict of interest.

## Abbreviations

OP: Obesity paradox
CVD: Cardiovascular disease
BMI: Body mass index
T2DM: Type 2 diabetes mellitus
ASCVD: Atherosclerotic cardiovascular disease
VFA: Visceral fat area
MMC: Metabolic Management Centre
HOMA-β: Homeostasis model assessment of β-cell function
FINS: Fasting serum insulin
FPG: Fasting plasma glucose
HOMA-IR: Homeostasis model assessment of insulin resistance
SFA: Subcutaneous fat area
SBP: Systolic blood pressure
DBP: Diastolic blood pressure
LDL-C: Low-density lipoprotein cholesterol
TC: Total cholesterol
OR: Odd ratio
ROC: Receiver operating characteristic
AUC: Area under the ROC curve
HbA_1c_: Glycosylated haemoglobin A_1c_
2h-PPG: 2 h postprandial plasma glucose
2h-PINS: 2 h postprandial serum insulin
FCP: Fasting C-peptide
2h-PCP: 2 h postprandial C-peptide
TG: Triglyceride
HDL-C: High-density lipoprotein cholesterol
Cr: Creatinine
ALT: Alanine aminotransferase
AST: Aspartate aminotransferase
γ-GT: Gamma glutamyl-transpeptidase
CI: Confidence interval
CT: Computed tomography
MRI: Magnetic resonance imaging
PCEs: Pooled Cohort Equations
