# Supplementary material for: Normal-weight visceral obesity promotes a higher 10-year atherosclerotic cardiovascular disease risk in patients with type 2 diabetes mellitus–a multicenter study in China
Source: Cardiovasc Diabetol. 2023 Jun 12;22:137. doi: 10.1186/s12933-023-01876-7 (PMC10262529; doi:10.1186/s12933-023-01876-7)
Supplement: Supplementary file 1 — Additional file 1: Figure S1 The proportion of high 10-year ASCVD risk according to BMI/VFA status in male patients with T2DM. Figure S2. The proportion of high 10-year ASCVD risk according to BMI/VFA status in female patients with T2DM. Table S1. Association between the other variables and VFA in T2DM patients. Table S2. Linear regression analysis of VFA in T2DM patients [file 12933_2023_1876_MOESM1_ESM.docx]

**SUPPLEMENTAL MATERIALS**


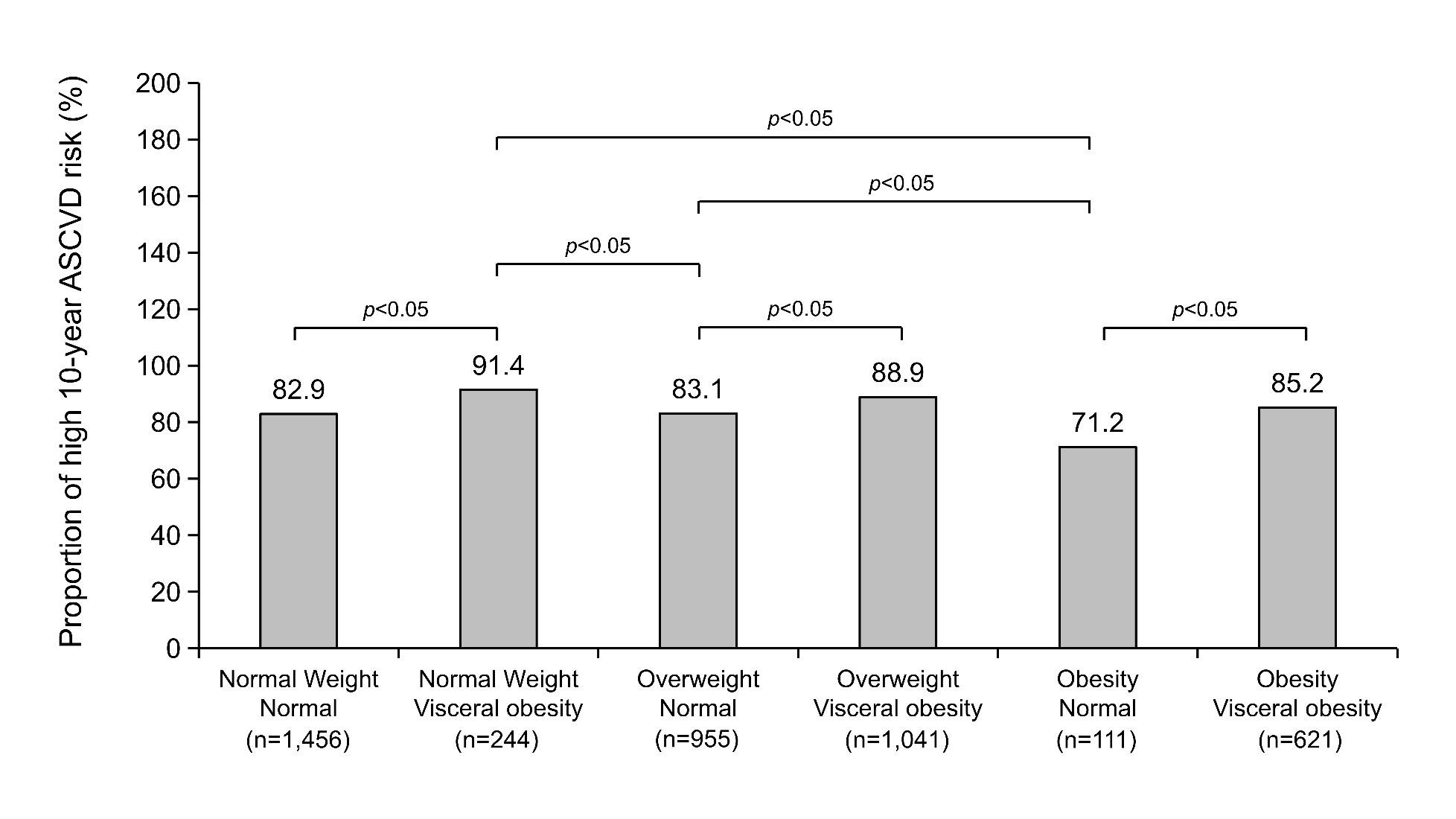


**Supplemental Fig.1** The proportion of high 10-year ASCVD risk according to BMI/VFA status in male T2DM patients. Patients were considered to have normal weight when 18.5 kg/m^2^ ≤ BMI < 24 kg/m^2^; overweight when 24 kg/m^2^ ≤ BMI < 28 kg/m^2^; and obesity when BMI ≥ 28 kg/m^2^. Visceral obesity was defined as VFA ≥ 100 cm^2^


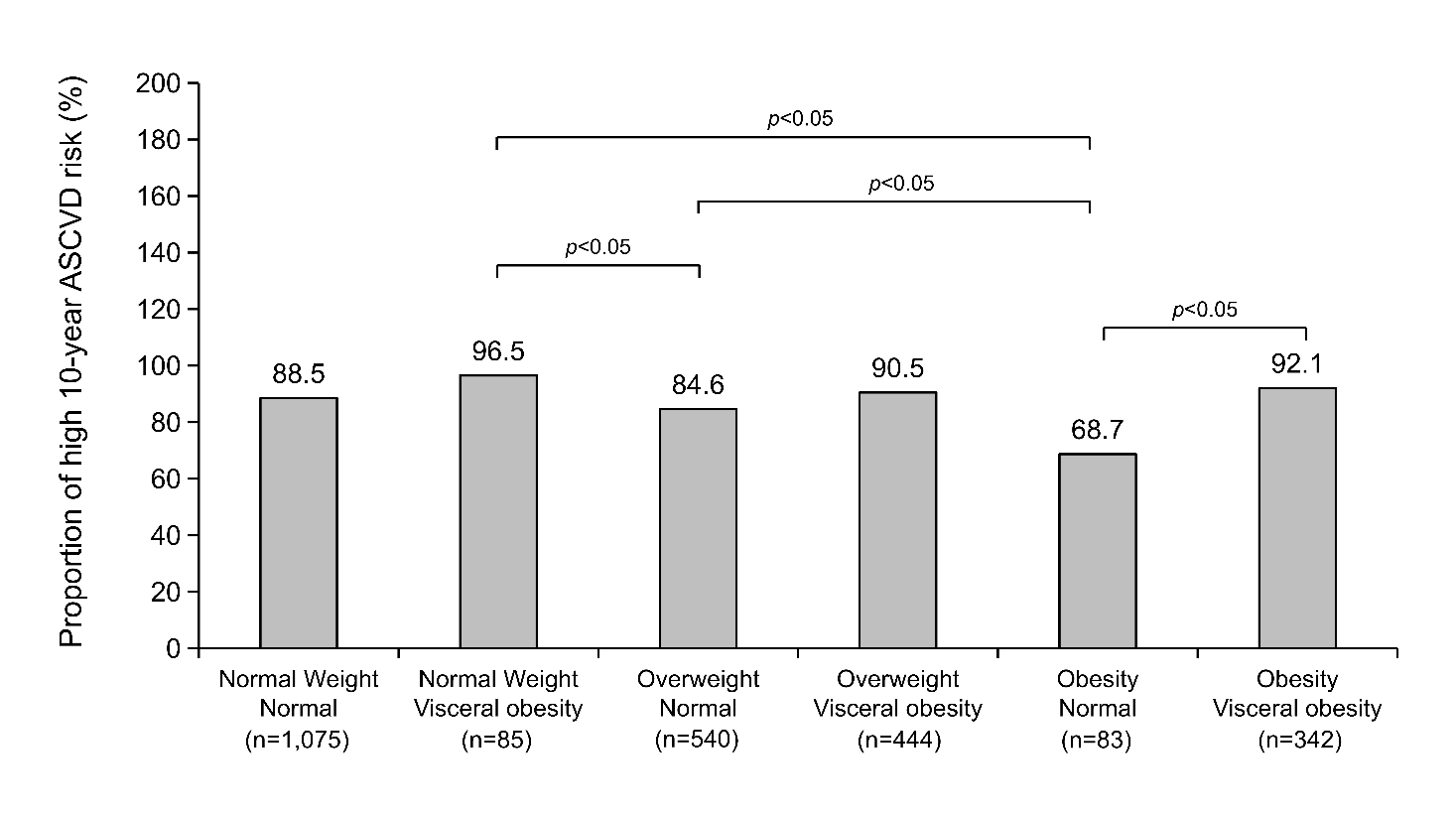


**Supplemental Fig.2** The proportion of high 10-year ASCVD risk according to BMI/VFA status in female T2DM patients. Patients were considered to have normal weight when 18.5 kg/m^2^ ≤ BMI < 24 kg/m^2^; overweight when 24 kg/m^2^ ≤ BMI < 28 kg/m^2^; and obesity when BMI ≥ 28 kg/m^2^. Visceral obesity was defined as VFA ≥ 100 cm^2^

**Supplemental Table 1** Association between the other variables and VFA in T2DM patients

|  | **R value** | ***P* value** |
| --- | --- | --- |
| **Male** | 0.096 | < 0.001 |
| **Age** | 0.050 | < 0.001 |
| **BMI** | 0.692 | < 0.001 |
| **VFA** | Null | Null |
| **SFA** | 0.674 | < 0.001 |
| **Hypertension** | 0.224 | < 0.001 |
| **Smoking** | 0.074 | < 0.001 |
| **Drinking** | 0.127 | < 0.001 |
| **Family history of diabetes** | -0.008 | 0.504 |
| **HbA_1c_** | -0.020 | 0.092 |
| **FBG** | 0.038 | 0.002 |
| **2h-PBG** | -0.034 | 0.006 |
| **FINs** | 0.223 | < 0.001 |
| **2h-PINs** | 0.186 | < 0.001 |
| **FCP** | 0.322 | < 0.001 |
| **2h-PCP** | 0.276 | < 0.001 |
| **HOMR-IR** | 0.207 | < 0.001 |
| **HOMR-β** | 0.161 | < 0.001 |
| **TG** | 0.314 | < 0.001 |
| **TC** | 0.116 | < 0.001 |
| **HDL-C** | -0.208 | < 0.001 |
| **LDL-C** | 0.079 | < 0.001 |

**Supplemental Table 2** Unilinear regression analysis of VFA in T2DM patients

|  | **R** | **R2** | **Adjusted R2** | **B** | **SE** | **β** | **t** | **Sig.** |
| --- | --- | --- | --- | --- | --- | --- | --- | --- |
| **Male** | 0.080 | 0.006 | 0.006 | 6.876 | 1.025 | 0.080 | 6.707 | < 0.001 |
| **Age** | 0.055 | 0.003 | 0.003 | 0.202 | 0.044 | 0.055 | 4.573 | < 0.001 |
| **Hypertension** | 0.224 | 0.050 | 0.050 | 18.714 | 0.972 | 0.224 | 19.257 | < 0.001 |
| **Smoking** | 0.068 | 0.005 | 0.004 | 6.016 | 1.058 | 0.068 | 5.688 | < 0.001 |
| **Drinking** | 0.125 | 0.016 | 0.015 | 10.838 | 1.113 | 0.125 | 9.742 | < 0.001 |
| **FBG** | 0.012 | < 0.001 | < 0.001 | -0.117 | 0.120 | -0.012 | -0.976 | 0.329 |
| **PBG** | 0.049 | 0.002 | 0.002 | -0.358 | 0.090 | -0.049 | -3.974 | < 0.001 |
| **FINs** | 0.066 | 0.004 | 0.004 | 0.105 | 0.021 | 0.066 | 5.097 | < 0.001 |
| **PINs** | 0.063 | 0.004 | 0.004 | 0.030 | 0.006 | 0.063 | 4.832 | < 0.001 |
| **FCP** | 0.288 | 0.083 | 0.083 | 8.157 | 0.332 | 0.288 | 24.587 | < 0.001 |
| **PCP** | 0.180 | 0.032 | 0.032 | 1.505 | 0.102 | 0.180 | 14.685 | < 0.001 |
| **HOMR-IR** | 0.053 | 0.003 | 0.003 | 0.194 | 0.047 | 0.053 | 4.092 | < 0.001 |
| **TG** | 0.209 | 0.044 | 0.044 | 4.756 | 0.266 | 0.209 | 17.879 | < 0.001 |
| **TC** | 0.103 | 0.011 | 0.011 | 3.368 | 0.387 | 0.103 | 8.700 | < 0.001 |
| **HDL-C** | 0.189 | 0.036 | 0.035 | -20.859 | 1.298 | -0.189 | -16.076 | < 0.001 |
| **LDL-C** | 0.063 | 0.004 | 0.004 | 2.496 | 0.473 | 0.063 | 5.279 | < 0.001 |

Abbreviations: SE, stand error; Sig, significance
